# Association between estimated glucose disposal rate control level and stroke incidence in middle‐aged and elderly adults

**DOI:** 10.1111/1753-0407.13595

**Published:** 2024-08-13

**Authors:** Jiangnan Yao, Feng Zhou, Lingzhi Ruan, Yiling Liang, Qianrong Zheng, Jiaxin Shao, Fuman Cai, Jianghua Zhou, Hao Zhou

**Affiliations:** ^1^ College of Nursing Wenzhou Medical University Wenzhou China; ^2^ Department of Global Health, School of Public Health Wuhan University of Science and Technology Wuhan China; ^3^ Department of Clinical Medicine Wenzhou Medical University Wenzhou China; ^4^ Department of Cardiology The First Affiliated Hospital of Wenzhou Medical University Wenzhou China

**Keywords:** CHARLS, estimated glucose disposal rate, stroke

## Abstract

**Background:**

To estimate glucose disposal rate (eGDR) as a newly validated surrogate marker of insulin resistance. Few studies have explored the association between changes in eGDR levels and stroke incidence. This study aims to explore the effect of the level of eGDR control on stroke and events.

**Methods:**

Data were obtained from the China Longitudinal Study on Health and Retirement (CHARLS). The eGDR control level was classified using K‐means cluster analysis. Logistic regression analysis was used to explore the association between different eGDR control levels and incident stroke. Restrictive cubic spline regression was used to test the potential nonlinear association between cumulative eGDR and stroke incidence.

**Results:**

Of the 4790 participants, 304 (6.3%) had a stroke within 3 years. The odds ratio (OR) was 2.34 (95% confidence interval [CI], 1.42–3.86) for the poorly controlled class 4 and 2.56 (95% CI, 1.53–4.30) for the worst controlled class 5 compared with class 1 with the best controlled eGDR. The OR for well‐controlled class 2 was 1.28 (95% CI, 0.79–2.05), and the OR for moderately controlled class 3 was 1.95 (95% CI, 1.14–3.32). In restrictive cubic spline regression analysis, eGDR changes are linearly correlated with stroke occurrence. Weighted quartile and regression analysis identified waist circumference and hypertension as key variables of eGDR for predicting incident stroke.

**Conclusions:**

Poorly controlled eGDR level is associated with an increased risk of stroke in middle‐aged and elderly people. Monitoring changes in eGDR may help identify individuals at high risk of stroke early.

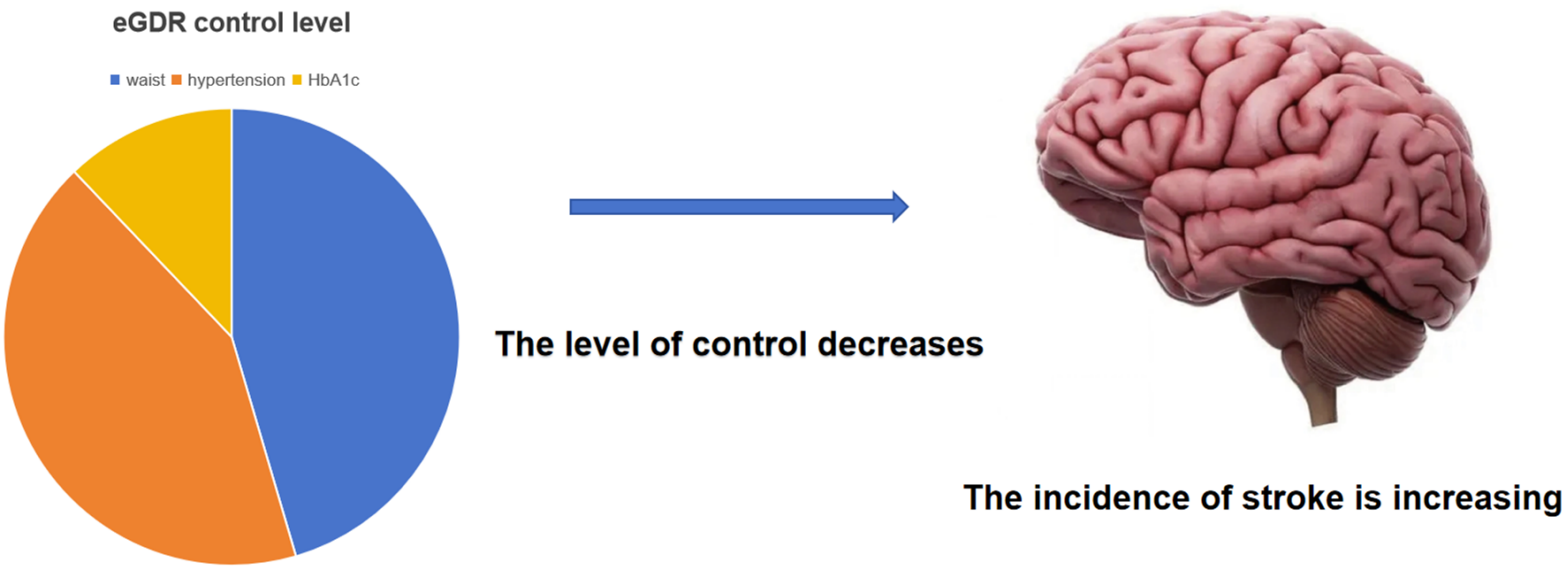

## Graphical abstract

Highlights

- In middle‐aged and elderly adults, poorly managed estimate glucose disposal rate (eGDR) levels are linked to an increased risk of stroke.
- As a result, controlling eGDR levels in stroke prevention measures needs additional focus.
- Furthermore, dynamic tracking of long‐term eGDR changes may be able to identify those who are more likely to have a stroke early on, which could make it easier to put more focused preventive measures in place.

## 1 INTRODUCTION

Stroke exerts a substantial health impact on a global scale. In 2019, stroke caused 143.23 million disability‐adjusted life years (DALYs) and resulted in 6.55 million deaths worldwide.^1^ While there have been improvements in medication and rehabilitation treatments, the burden of disease (measured in DALYs) and the fatality rates from stroke have reduced in high‐income nations over the past few decades. Nevertheless, the prevalence of stroke has increased significantly in nations with lower income and lower middle income, contributing to 89.0% of DALYs and 86.0% of deaths.^2^ Therefore, it is critical to develop cost‐effective and reproducible indicators to identify individuals with high risk of stroke.

Insulin resistance (IR) is defined as the need for higher doses of insulin to elicit a quantitatively normal response. Current research indicates that IR may increase the risk of stroke by causing atherosclerosis. For determining insulin sensitivity, the hyperinsulinemia‐euglycemic clamp test has long been considered the “gold standard” method.^3^, ^4^ However, its application in clinical practice is expensive and complicated to operate.^5^ Therefore, alternative noninvasive testing approaches, such as triglyceride glucose index, homeostasis model assessment‐IR, and estimated glucose disposal rate (eGDR), are receiving more attention. The eGDR control is a straightforward and noninvasive method for evaluating IR. It involves three clinically accessible variables: hypertension, waist circumference, and glycated hemoglobin A (HbA1c).^6^ Previous studies showed that eGDR was accurate for assessing IR, and low eGDR was significantly associated with an increased risk of mortality in diabetic individuals.^7^

Some studies also explored the predictive performance of eGDR in cardiovascular diseases. A nationwide population‐based observational cohort study of 104 697 persons diagnosed with type 2 diabetes found that individuals with low eGDR were associated with a one‐third decreased risk of stroke.^8^ In addition, another study with 6 years of follow‐up found that each 1‐SD increase of eGDR was associated with a 16% decreased risk of cerebrovascular disease (CVD).^9^ These findings indicated that eGDR was independent of traditional cardiovascular predictors.^10^ Although previous studies have provided insightful information, most studies have focused on baseline eGDR values, and studies investigating the association between altered eGDR and stroke incidence have not been documented in the literature. In addition, blood glucose values are constantly fluctuating, and the degree of glycemic control varies among different populations. Our study is to analyze data from the China Longitudinal Study on Health and Retirement (CHARLS) in order to ascertain the impact of eGDR control level on the occurrence of stroke.

## 2 METHODS

### 2.1 Study population

CHARLS is a long‐term follow‐up survey in China to study the health, retirement, and economic status of elderly Chinese.^11^ The Chinese Academy of Social Sciences, Peking University, and other institutions collaborated to organize and conduct the CHARLS survey. The survey conducted the first baseline study in 2011, the second wave in 2013, the third wave in 2015, and the fourth wave in 2018. Blood samples were collected during the baseline and third waves.^12^ To be eligible for this analysis, participants had to be at least 45 years old and provide complete information on their body mass index (BMI), triglycerides (TG), and glucose. Those who had experienced a stroke before 2015 were not included in this study. Following the baseline survey, all individuals were followed up every 2 years. The Biomedical Ethics Review Committee of Peking University (IRB000010052‐11,015) authorized the ethical application for the collection of CHARLS human subject data, and it was carried out in compliance with the Declaration of Helsinki. Furthermore, formal informed consent was supplied by each CHARLS participant. Information about the CHARLS data is available on their website (http://charls.pku.edu.cn/en).

### 2.2 Data assessment

#### 2.2.1 The change in eGDR and stroke

The main focus of this study is to have the occurrence of stroke as its primary outcome. “Have you been diagnosed with a stroke?” Stroke patients are those who answered “yes” to this question.^13^, ^14^

This study concentrated on the differences in eGDR obtained between 2012 and 2015. IR was measured by eGDR (mg/kg/min), which was first computed using the following formula: eGDR is equal to 21.158− (0.09 × WC) − (3.407 × HT) − (0.551 × HbA1c), where HT = hypertension (yes = 1/no = 0), WC = waist circumference (cm), and HbA1c = HbA1c (%). The measurement of waist circumference was performed using the natural waist circumference position. Blood pressure ≥140/90 mmHg twice or the use of antihypertensive drugs was considered hypertension. Venous blood samples were taken from the participants to determine the HbA1c, which is then expressed as a percentage in the formula.^9^ The formula for cumulative eGDR change is (eGDR_2012_ + eGDR_2015_)/2 × time (2015–2012).

#### 2.2.2 Data collection

In the first wave of the study, trained interviewers used structured questionnaires to collect data on blood pressure readings, waist circumference, BMI, blood pressure‐related factors (age, sex, HuKou, education, and marital status), and potential risk factors (diabetes, dyslipidemia, heart problems, smoking status, and alcohol consumption). Primary prevention includes lipid lowering and treatment for hypoglycemia. The following are examined in a lab setting: blood urea nitrogen (BUN), glucose, triglycerides (TG), total cholesterol (TC), high‐density lipoprotein cholesterol (HDL‐C), low‐density lipoprotein cholesterol (LDL‐C), glycated hemoglobin, C‐reactive protein (CRP), and uric acid (UA).^15^

### 2.3 Statistical analysis

**FIGURE 1:**
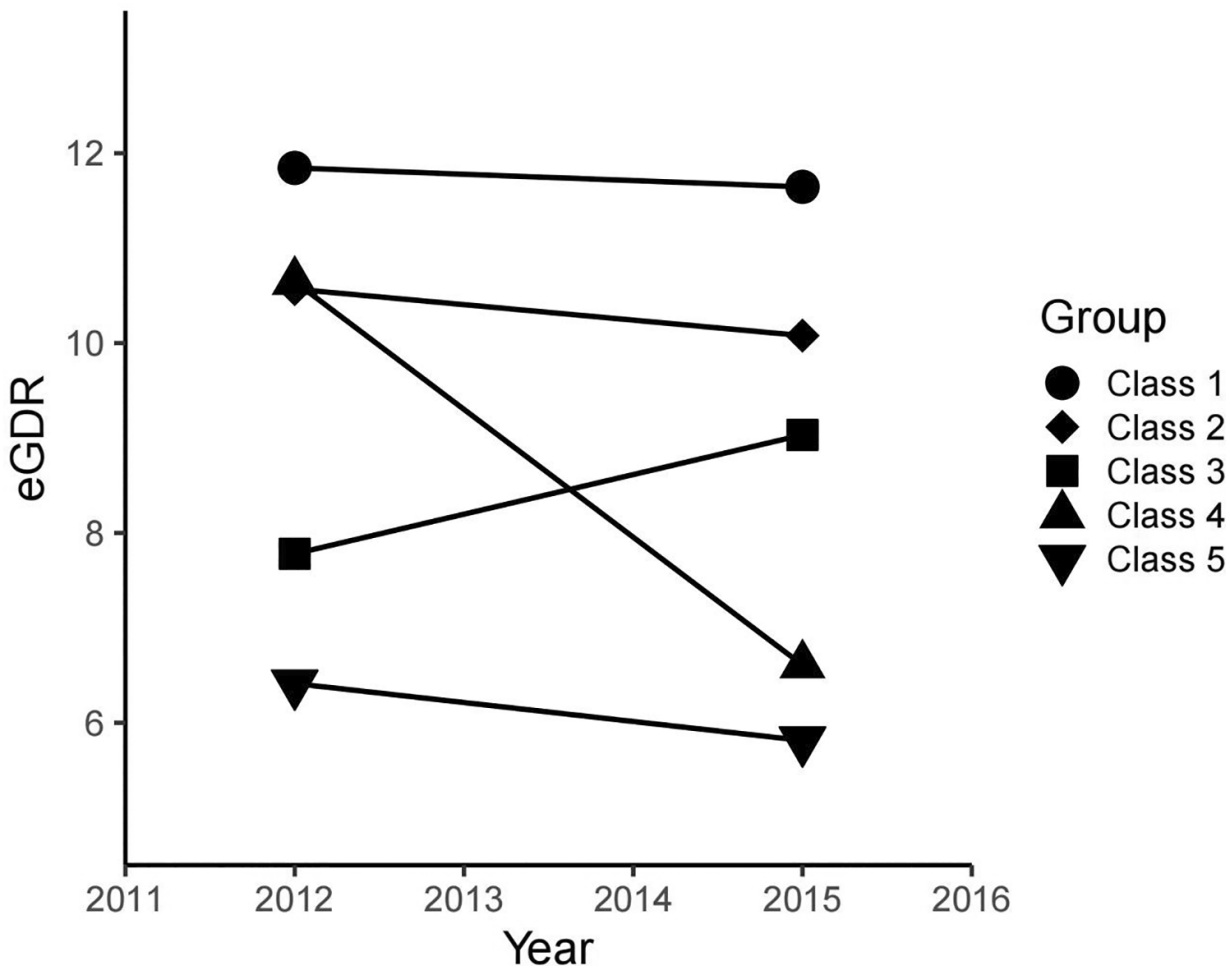
The eGDR clustering by K‐means clustering.

K‐means clustering is a technique for dividing *N* observations into *K* clusters.^16^ The K‐means clustering method was used to analyze the 3‐year transition dataset of eGDR and divided the observations into five categories. Each observation is assigned to the cluster with the closest mean as a prototype for clustering.^17^ The maximum number of classes recruited is five, and each class contains no less than two data. When the number of clusters is five, K‐means clustering outperforms other methods. The final classification was made for each of the five categories that met the following requirements: class 1 observed a slight decrease in eGDR from 11.85 in 2012 to 11.65 in 2015, indicating best eGDR control; class 2 observed that eGDR decreased from 10.57 in 2012 to 10.08 in 2015, indicating that eGDR control well. An increase in eGDR from 7.78 in 2012 to 9.03 in 2015 was observed in class 3, indicating moderate eGDR control; a significant decrease from 10.65 in 2012 to 6.61 in 2015 was observed in class 4, indicating poor eGDR control; and class 5 observed a decrease in eGDR from 6.41 in 2012 to 5.81 in 2015, indicating that eGDR was worst controlled (Figure 1).

Descriptive statistics, including means and standard deviations for continuous variables and percentages for categorical variables, were used to illustrate the essential features of the research population. For categorical variables, the chi‐square test or Fisher's exact test was employed, and for baseline characteristic differences across groups, the *t*‐test or Mann–Whitney *U* test was utilized. Using univariate and multivariate logistic regression analyses, significant variables related to the incidence of stroke were identified. A constrained cubic spline model was also used to examine the relationship between cumulative eGDR and stroke, with a focus on the form of the connection. In order to rule out any confounding variables, we also conducted a subgroup analysis to look into the relationship between eGDR variants and stroke.

A mathematical model integrating the variables waist circumference, hypertension, and HbA1c was used to derive eGDR. Diastolic blood pressure (DBP), systolic blood pressure (SBP), and hypertension medicines typically control the hypertension variable. When evaluating and managing the health of patients with hypertension, these criteria are frequently taken into consideration in clinical practice or scientific study. We used a regression model called weighted quantile sum (WQS) in order to fully explain the equation.^18^ We used bootstrap resampling methods, repeating the process 1000 times, to adjust for variability. We were able to weigh HbA1c, hypertension, and waist circumference using the WQS model, estimating how much each contributed to the overall effect. These weights were constrained within the range of 0–1, with a cumulative sum of 1.^19^ Higher weights were assigned to indicate the higher significance of the corresponding indicator in predicting stroke. The relationship between changes in eGDR and stroke was expressed in odds ratios (ORs) (95% confidence interval [CI]). Statistical analysis was performed using the software R version 4.1.0. (http://www.R-project.org/). All test were two‐tailed, and statistical significance was defined as *p* < 0.05.

## 3 RESULTS

### 3.1 Baseline characteristics of study participants

**FIGURE 2:**
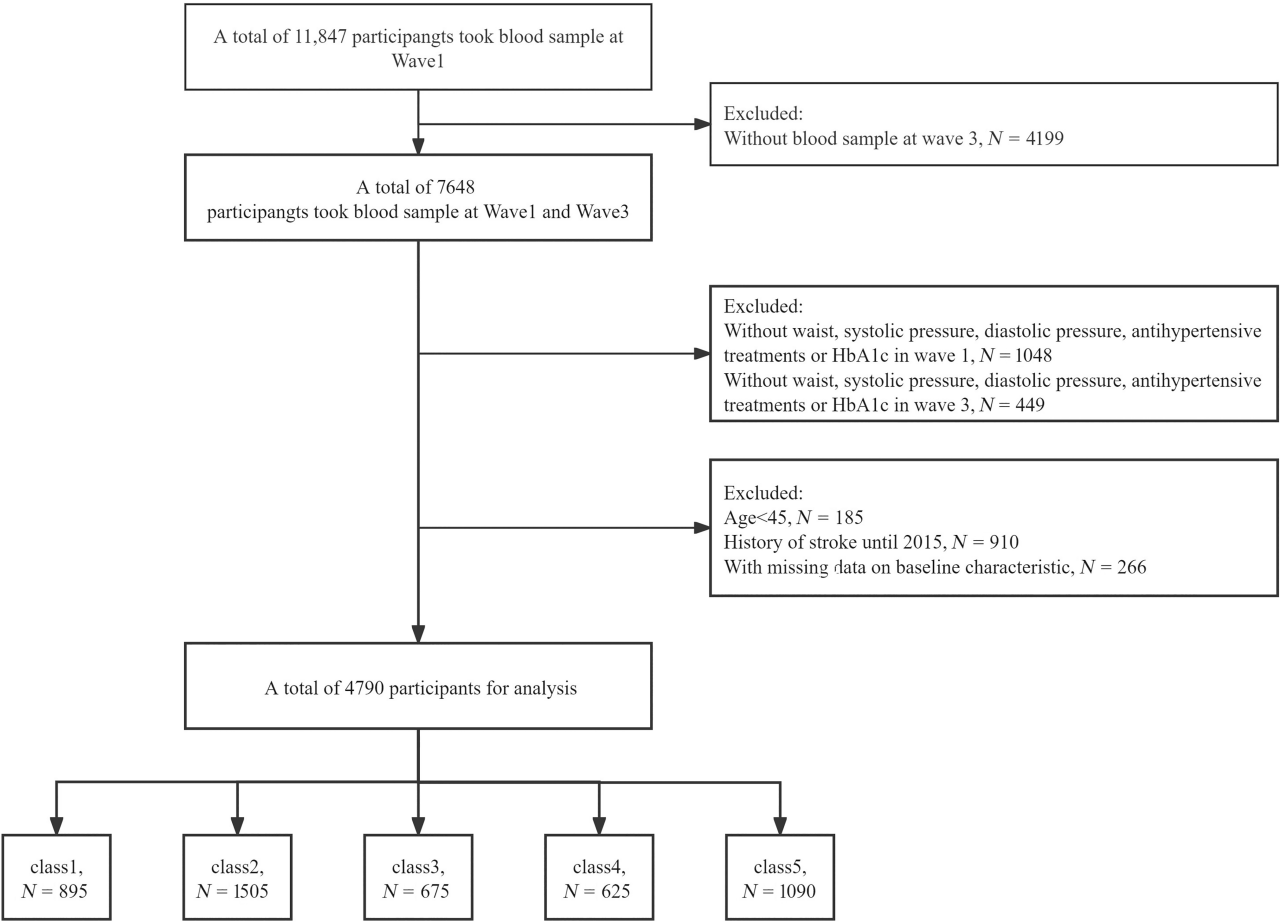
Flowchart of the study population.

Of the 11 847 participants at the study baseline, we excluded 4199 individuals who lacked blood sample data at wave 3. In addition, 1497 participants were excluded due to their absence of lumbar pressure and DBP, antihypertensive therapy, or incomplete information on HbA1c at waves 1 and 3. In addition, we excluded 185 participants younger than 45 years, 910 individuals who reported a history of stroke at waves 1 and 3, and 266 participants who lacked baseline trait data. In the end, a total of 4790 participants met the inclusion criteria and were included in subsequent analyses (Figure 2). The study involved participants averaging 58.3 ± 8.4 years old, with 44.6% being male. In 2012, the mean eGDR was 9.5 ± 2.2, which decreased to 8.8 ± 2.4 in 2015. The average cumulative eGDR was 27.4 ± 6.3. Using the group with the highest average eGDR as the control group, we found that participants in other categories had lower rates of smoking and drinking, higher BMI, SBP, and DBP, and higher prevalence of diseases such as hypertension, diabetes, dyslipidemia, and heart disease. They also showed higher levels of glucose, TG, TC, LDL, HbA1c, and UA, and lower levels of HDL compared with those in class 1 (Table 1).

**TABLE 1:** Characteristics of the study according to the change in eGDR.

Characteristics	Total	Class 1	Class 2	Class 3	Class 4	Class 5	*p* value
*N* (%)	4790	895	1505	675	625	1090	
Age, years	58.3 ± 8.4	57.7 ± 8.5	56.6 ± 7.8	60.1 ± 9.2	58.6 ± 8.4	59.9 ± 8.3	<0.001
Gender (male) (%)	2137 (44.6%)	417 (46.6%)	658 (43.7%)	320 (47.4%)	301 (48.2%)	441 (40.5%)	0.005
Education level (%)							
Primary school or lower	3367 (70.3%)	650 (72.6%)	1001 (66.5%)	496 (73.5%)	444 (71.0%)	776 (71.2%)	0.002
Secondary school or higher	1423 (29.7%)	245 (27.4%)	504 (33.5%)	179 (26.5%)	181 (29.0%)	314 (28.8%)	
Current married (%)	4322 (90.2%)	818 (91.4%)	1382 (91.8%)	589 (87.3%)	566 (90.6%)	967 (88.7%)	0.004
HuKou (%)							
Agriculture	4115 (85.9%)	811 (90.6%)	1296 (86.1%)	574 (85.0%)	533 (85.3%)	901 (82.7%)	<0.001
Others	675 (14.1%)	84 (9.4%)	209 (13.9%)	101 (15.0%)	92 (14.7%)	189 (17.3%)	
Smoking (%)	1421 (29.7%)	306 (34.2%)	431 (28.6%)	231 (34.2%)	186 (29.8%)	267 (24.5%)	<0.001
Drinking (%)	1566 (32.7%)	328 (36.6%)	487 (32.4%)	234 (34.7%)	221 (35.4%)	296 (27.2%)	<0.001
SBP, mmHg	128.0 ± 20.7	114.3 ± 11.9	116.7 ± 11.8	142.2 ± 19.5	125.3 ± 11.9	147.8 ± 20.1	<0.001
DBP, mmHg	74.8 ± 12.0	67.4 ± 8.7	70.0 ± 9.0	81.3 ± 11.5	74.0 ± 9.0	84.1 ± 12.1	<0.001
Waist circumference (SD)	84.5 ± 12.2	72.4 ± 11.6	85.4 ± 7.0	85.7 ± 10.4	83.1 ± 15.3	93.6 ± 8.5	
BMI, kg/m^2^	24.2 ± 35.9	20.4 ± 2.6	23.8 ± 12.2	23.6 ± 4.3	27.7 ± 97.1	26.3 ± 3.9	<0.001
Diabetes (%)	280 (5.8%)	11 (1.2%)	38 (2.5%)	47 (7.0%)	38 (6.1%)	146 (13.4%)	<0.001
Heart problems (%)	526 (11.0%)	59 (6.6%)	124 (8.2%)	67 (9.9%)	71 (11.4%)	205 (18.8%)	<0.001
Dyslipidemia (%)	470 (9.8%)	23 (2.6%)	111 (7.4%)	56 (8.3%)	55 (8.8%)	225 (20.6%)	<0.001
Diabetes treatments (%)	147 (3.1%)	4 (0.4%)	19 (1.3%)	24 (3.6%)	19 (3.0%)	81 (7.4%)	<0.001
Heart problem treatments (%)	310 (6.5%)	37 (4.1%)	63 (4.2%)	50 (7.4%)	38 (6.1%)	122 (11.2%)	<0.001
Lipid‐lowering treatments (%)	231 (4.8%)	8 (0.9%)	44 (2.9%)	30 (4.4%)	19 (3.0%)	130 (11.9%)	<0.001
Glucose, mg/dL	109.3 ± 34.5	100.6 ± 17.1	103.6 ± 19.6	114.5 ± 45.4	110.0 ± 33.6	120.6 ± 47.9	<0.001
Triglycerides, mg/dL	132.1 ± 96.5	97.7 ± 55.7	124.1 ± 83.3	134.6 ± 100.2	138.5 ± 103.1	166.3 ± 119.4	<0.001
BUN, mg/dL	15.6 ± 4.3	15.9 ± 4.5	15.5 ± 4.2	16.1 ± 4.7	15.5 ± 4.4	15.5 ± 4.1	0.021
TC, mg/dL	193.3 ± 37.6	185.7 ± 35.2	191.0 ± 35.9	195.7 ± 36.0	195.5 ± 39.5	200.2 ± 40.2	<0.001
HDL‐C, mg/dL	51.2 ± 15.3	57.5 ± 15.5	50.9 ± 14.1	52.5 ± 16.5	51.1 ± 15.6	45.9 ± 13.6	<0.001
LDL‐C, mg/dL	116.2 ± 34.4	110.5 ± 30.4	116.3 ± 32.5	116.1 ± 34.2	116.7 ± 35.0	120.7 ± 38.9	<0.001
CRP, mg/L	2.5 ± 7.0	2.1 ± 8.2	2.3 ± 7.4	2.6 ± 6.6	2.4 ± 5.6	2.9 ± 6.1	<0.001
HbA1c, %(mmol/mol)	5.3 ± 0.8	5.0 ± 0.4	5.2 ± 0.4	5.4 ± 1.0	5.2 ± 0.7	5.5 ± 1.1	<0.001
UA, mg/dL	4.3 ± 1.2	4.1 ± 1.1	4.3 ± 1.1	4.4 ± 1.2	4.4 ± 1.3	4.6 ± 1.3	<0.001
eGDR_2012_	9.5 ± 2.2	11.8 ± 1.1	10.6 ± 0.6	7.8 ± 0.9	10.6 ± 1.1	6.4 ± 1.0	<0.001
eGDR_2015_	8.8 ± 2.4	11.6 ± 1.2	10.1 ± 0.7	9.0 ± 1.4	6.6 ± 1.0	5.8 ± 1.1	<0.001
Cumulative eGDR	27.4 ± 6.3	35.2 ± 2.1	31.0 ± 1.5	25.2 ± 2.1	25.9 ± 2.6	18.3 ± 2.7	<0.001
Incidence of stroke (%)	304 (6.3%)	26 (2.9%)	58 (3.9%)	50 (7.4%)	48 (7.7%)	122 (11.2%)	<0.001

Abbreviations: BMI, body mass index; BUN, blood urea nitrogen; CRP, C‐reactive protein; DBP, diastolic blood pressure; eGDR, estimated glucose disposal rate; HbA1c, hemoglobin A1C; HDL‐C, high‐density lipoprotein cholesterol; LDL‐C, low‐density lipoprotein cholesterol; SBP, systolic blood pressure; TC, total cholesterol; UA, uric acid.

### 3.2 Odds ratios for incident stroke

**FIGURE 3:**
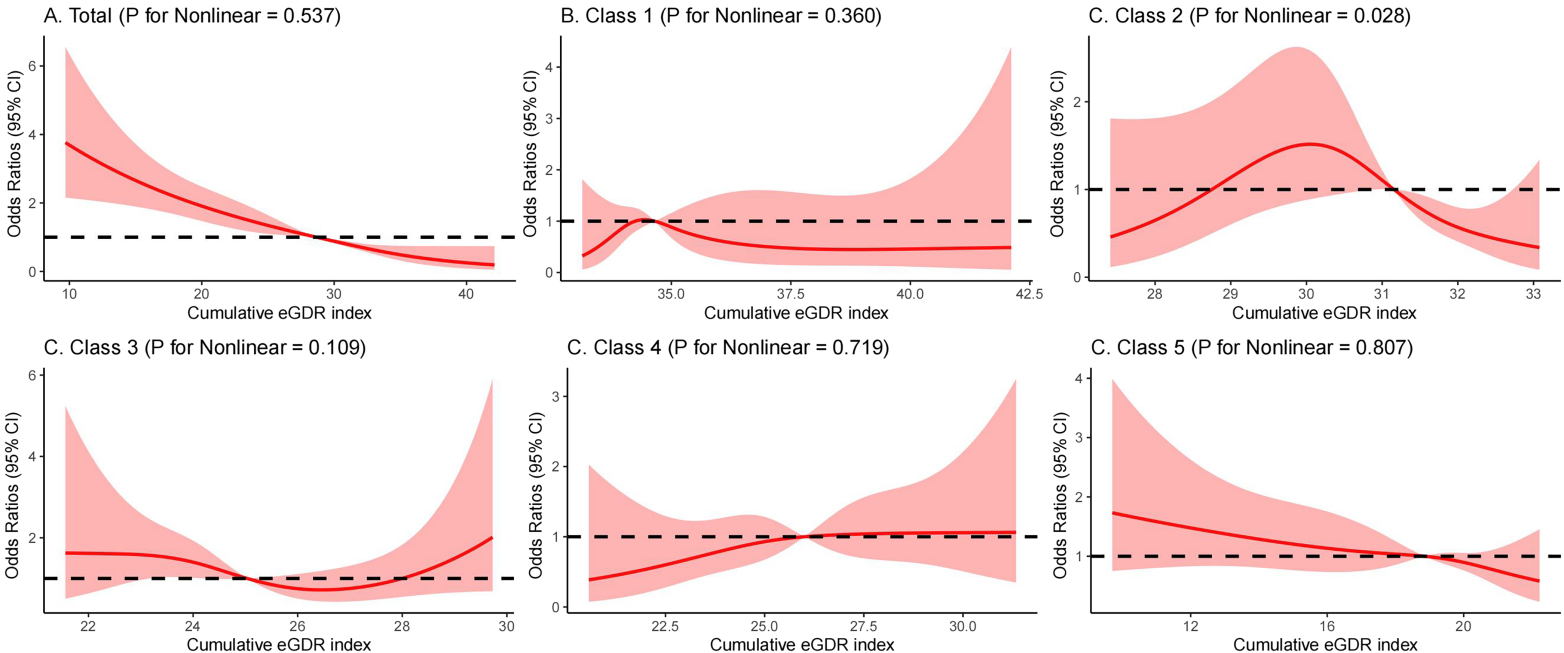
Cubic model of the association between different classes and cumulative eGDR.

Three years later, 304 people (6.3%) in the study had suffered a stroke. The OR for stroke in class 2 was 1.34 (95% CI, 0.84–2.14) compared with class 1. Class 3 and Class 4 have similar eGDR, but the trend is different, with the risk of stroke higher in class 4 than in class 3. The OR was 2.67 (95% CI, 1.65–4.34) for class 3 and 2.78 (95% CI, 1.71–4.53) for class 4. Class 5 eGDR was the worst controlled and had the highest risk of new stroke, with an OR of 4.21 (95% CI, 2.73–6.50). After adjusting for various factors such as age, gender, education, marital status, HuKou (household registration), smoking status, drinking status, SBP, waist circumference, BMI and history of diabetes mellitus, heart disease, dyslipidemia, hypoglycemic treatments, heart problem treatments, and lipid‐lowering treatments (Model 3), compared with class 1, the OR was 1.28 (95% CI, 0.79–2.05) for class 2, 1.95 (95% CI, 1.14–3.32) for class 3, 2.34 (95% CI, 1.42–3.86) for class 4, and 2.56 (95% CI, 1.53–4.30) for class 5. We found no increased risk of stroke from class 1 to class 5 (Table 2). The restricted cubic spline regression model showed a linear relationship between cumulative eGDR and the risk of stroke, as depicted in Figure 3 (*p* = 0.537). The risk of stroke gradually decreased as the cumulative eGDR exceeded 28.59 (OR, 0.99; 95% CI, 0.98–1.00). Participants had a reduced likelihood of experiencing a stroke as the cumulative eGDR surpassed 31.14 (OR, 0.99; 95% CI, 0.98–1.00) in the second category and 18.82 (OR, 1.00; 95% CI, 0.99–1.01) in the fifth category. Meantime, after the incidence of stroke was classified by quintile of cumulative mean eGDR, the incidence of stroke gradually decreased from Q1 to Q5 in the five groups, and 110 (11.5%), 72 (7.5%), 62 (6.5%), 33 (3.4%), and 27 (2.8%) were observed in the five groups of participants, respectively. After adjusting for multiple covariates, the adjusted logistic regression model showed that higher levels of cumulative mean eGDR reduced the odds ratio for stroke events (Table S1).

**TABLE 2:** Logistic regression analysis for the association between different classes and stroke.

Cluster	Crude	Model I	Model II	Model III
	OR (95% CI); *p* value	OR (95% CI); *p* value	OR (95% CI); *p* value	OR (95% CI); *p* value
Class 1	Reference	Reference	Reference	Reference
Class 2	1.34 (0.84, 2.14); 0.223	1.37 (0.85, 2.19); 0.191	1.36 (0.84, 2.17); 0.208	1.28 (0.79, 2.05); 0.315
Class 3	2.67 (1.65, 4.34); <0.001	2.54 (1.56, 4.13); <0.001	2.26 (1.33, 3.83); 0.003	1.95 (1.14, 3.32); 0.015
Class 4	2.78 (1.71, 4.53); <0.001	2.73 (1.68, 4.46); <0.001	2.61 (1.59, 4.30); <0.001	2.34 (1.42, 3.86); <0.001
Class 5	4.21 (2.73, 6.50); <0.001	4.01 (2.60, 6.20); <0.001	3.53 (2.14, 5.83); <0.001	2.56 (1.53, 4.30); <0.001

*Note*: Model I, adjusted for age, gender. Model II, adjusted for age, gender, education, marital status, Hukou, smoking status, drinking status, systolic blood pressure, diastolic blood pressure and body mass index. Model III, adjusted for factors in model II and history of diabetes, heart disease, dyslipidemia, hypoglycemic treatments, heart problems treatments and lipid‐lowering treatments.

### 3.3 Subgroup analyses

We performed a stratified analysis of the relationship between changes in eGDR levels over 3 years and stroke occurrence in participants, as shown in Table 3. The high‐risk factors for stroke are >60 years old, female, married, and agriculture HuKou. In patients without diabetes, dyslipidemia, or heart disease, the two were positively correlated. There was no significant interaction between eGDR level changes and the above subgroups.

**TABLE 3:** Subgroup analysis of the associations between different classes and stroke.

	Case	Class 1	Class 2	Class 3	Class 4	Class 5	*p* for interaction
Age (years)							
<54	1469	1.0	1.3 (0.6, 3.3), 0.508	0.9 (0.2, 3.3), 0.881	2.3 (0.9, 6.4), 0.099	2.4 (0.7, 7.9), 0.141	0.808
54–60	1319	1.0	1.1 (0.4, 3.0), 0.848	1.8 (0.6, 5.7), 0.287	2.5 (0.9, 7.0), 0.076	2.1 (0.7, 6.5), 0.205	
>60	2002	1.0	1.3 (0.6, 2.7), 0.446	2.4 (1.1, 5.0), 0.022	2.1 (1.0, 4.5), 0.048	2.8 (1.3, 5.7), 0.007	
Gender							
Male	2137	1.0	1.0 (0.5, 2.1), 0.917	1.7 (0.8, 3.8), 0.174	2.1 (1.0, 4.4), 0.042	1.8 (0.8, 4.0), 0.178	0.744
Female	2653	1.0	1.4 (0.7, 2.8), 0.290	2.0 (1.0, 4.3), 0.061	2.4 (1.2, 4.8), 0.017	3.0 (1.5, 6.2), 0.002	
Education level							
Primary school or lower	3367	1.0	1.0 (0.6, 1.7), 0.936	1.8 (1.0, 3.3), 0.040	2.0 (1.1, 3.4), 0.015	2.3 (1.3, 4.2), 0.004	0.293
Secondary school or higher	1423	1.0	4.3 (1.0, 18.8), 0.054	4.1 (0.8, 20.3), 0.081	6.7 (1.4, 30.7), 0.015	5.9 (1.3, 27.4), 0.025	
Current married							
No	468	1.0	0.9 (0.3, 2.7), 0.811	1.5 (0.4, 5.0), 0.514	1.1 (0.3, 4.2), 0.851	1.5 (0.4, 5.6), 0.572	0.752
Yes	4322	1.0	1.4 (0.8, 2.3), 0.257	2.0 (1.1, 3.7), 0.025	2.7 (1.6, 4.7), <0.001	2.9 (1.7, 5.2), <0.001	
HuKou							
Agriculture	4115	1.0	1.2 (0.7, 2.0) 0.459	2.0 (1.1, 3.4), 0.018	2.1 (1.2, 3.5) 0.006	2.4 (1.4, 4.1), 0.002	0.586
Others	675	1.0	2.6 (0.3, 22.8), 0.376	2.6 (0.3, 25.6), 0.416	7.1 (0.8, 60.8), 0.073	6.1 (0.7, 55.5), 0.108	
Current smoking status							
No	3369	1.0	1.4 (0.8, 2.6), 0.201	2.0 (1.1, 3.9), 0.031	2.4 (1.3, 4.5), 0.004	2.6 (1.4, 4.8), 0.003	0.831
Yes	1421	1.0	0.8 (0.3, 2.1), 0.700	1.7 (0.7, 4.5), 0.270	2.1 (0.8, 5.1), 0.117	2.5 (1.0, 6.4), 0.058	
Drinking status							
No	3224	1.0	1.5 (0.8, 2.7), 0.207	2.3 (1.2, 4.5), 0.017	2.1 (1.1, 4.1), 0.026	3.2 (1.7, 6.1), <0.001	0.261
Yes	1566	1.0	0.9 (0.4, 2.0), 0.814	1.3 (0.5, 3.1), 0.587	2.4 (1.1, 5.3), 0.029	1.4 (0.6, 3.6), 0.460	
Diabetes							
No	4510	1.0	1.3 (0.8, 2.1), 0.285	2.0 (1.2, 3.5), 0.013	2.4 (1.5, 4.1), <0.001	2.6 (1.5, 4.5), <0.001	0.935
Yes	280	1.0	0.6 (0.0, 7.2), 0.682	0.7 (0.1, 8.5), 0.773	0.7 (0.1, 8.8), 0.796	1.1 (0.1, 11.3), 0.959	
Heart problem							
No	4264	1.0	1.3 (0.8, 2.2), 0.334	1.9 (1.0, 3.4), 0.035	2.6 (1.5, 4.5), <0.001	2.4 (1.4, 4.3), 0.003	0.445
Yes	526	1.0	1.0 (0.3, 3.6), 0.962	1.5 (0.4, 5.9), 0.563	0.8 (0.2, 3.4), 0.786	1.7 (0.5, 6.4), 0.411	
Dyslipidemia							
No	4320	1.0	1.3 (0.8, 2.1), 0.330	2.0 (1.1, 3.5), 0.017	2.6 (1.6, 4.4), <0.001	2.5 (1.4, 4.3), 0.002	0.366
Yes	470	1.0	1.6 (0.2, 14.2), 0.655	2.5 (0.3, 23.0), 0.421	1.1 (0.1, 11.5), 0.946	3.4 (0.4, 29.1), 0.264	
Heart problem treatments							
No	4480	1.0	1.3 (0.8, 2.1), 0.355	1.8 (1.0, 3.1), 0.046	2.4 (1.4, 4.1), <0.001	2.4 (1.4, 4.1), 0.002	0.409
Yes	310	1.0	1.4 (0.3, 8.1), 0.689	3.3 (0.6, 19.7), 0.188	0.9 (0.1, 7.3), 0.920	3.3 (0.6, 17.2), 0.164	
SBP							
<117	1573	1.0	1.0 (0.5, 2.0), 0.928	2.2 (0.7, 7.1), 0.196	2.4 (1.0, 5.9), 0.057	3.2 (1.0, 9.8), 0.048	0.580
117–1334	1604	1.0	2.3 (1.0, 5.3), 0.054	2.6 (0.9, 7.6), 0.086	3.5 (1.5, 8.1), 0.005	2.9 (1.1, 7.7), 0.029	
>134	1613	1.0	0.6 (0.2, 2.6), 0.536	1.0 (0.3, 3.1), 0.946	0.9 (0.3, 3.1), 0.874	1.3 (0.4, 4.0), 0.610	
DBP							
<69	1552	1.0	1.3 (0.6, 2.7), 0.559	1.7 (0.5, 5.4), 0.389	4.7 (2.1, 10.4) <0.001	4.9 (1.8, 13.4), 0.002	0.245
69–79	1557	1.0	1.2 (0.5, 2.5), 0.682	2.4 (1.0, 6.0), 0.052	1.6 (0.7, 3.6), 0.309	2.3 (0.9, 5.7), 0.077	
>79	1681	1.0	1.6 (0.5, 5.9), 0.446	1.7 (0.5, 6.0), 0.384	2.1 (0.6, 7.5), 0.267	2.3 (0.7, 7.7), 0.192	
BMI							
<21.7	1583	1.0	1.1 (0.6, 2.3), 0.691	1.2 (0.5, 3.0), 0.650	2.8 (1.4, 5.7), 0.004	2.2 (0.8, 6.0), 0.124	0.649
21.7–24.8	1621	1.0	1.5 (0.5, 4.5), 0.458	3.6 (1.1, 11.8), 0.033	3.4 (1.1, 10.4), 0.034	3.5 (1.1, 11.4), 0.037	
>24.8	1586	1.0	1.1 (0.1, 8.9), 0.901	1.5 (0.2, 12.3), 0.686	1.3 (0.2, 10.5), 0.798	1.7 (0.2, 13.6), 0.598	

Abbreviations: BMI, body mass index; DBP, diastolic blood pressure; SBP, systolic blood pressure.

### 3.4 WQS analyses

**FIGURE 4:**
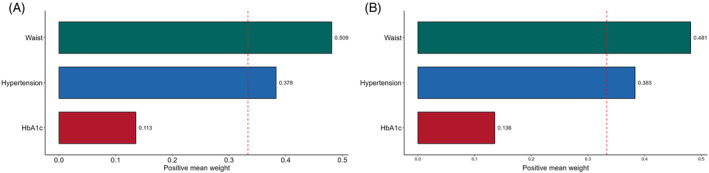
Estimated weights assigned to eGDR with the WQS model. Weights in a negative direction were obtained when the effect parameter of the WQS model was constrained to the negative direction with 1000 repeated holdout validations for eGDR in 2012 (A) and 2015 (B).

The WQS regression model was used to provide a detailed explanation of the eGDR, taking into account potential confounding variables and the cumulative impact of stroke. The weights assigned to the different components of eGDR are shown in Figure 4. In both 2012 and 2015, waist circumference and hypertension were identified as significant factors for stroke risk, with slight differences in their weights. Importantly, waist circumference emerged as the primary contributor in both 2012 and 2015, with weights of 0.509 and 0.481, respectively.

## 4 DISCUSSION

The current research discovered a strong correlation between a poorly controlled eGDR level and a higher likelihood of stroke in middle‐aged and elderly Chinese adults. This suggests that maintaining a consistently low eGDR level is a significant predictor of stroke occurrence. It is advisable to regularly monitor changes in eGDR over an extended period of time in individuals who are at a high risk of stroke, particularly in the initial stages. We have additionally proven a direct correlation between the cumulative eGDR and the occurrence of strokes, which follows a linear trend. Therefore, the eGDR score can be valuable in identifying patients with a heightened risk of stroke. Furthermore, we employed WQS regression to investigate the impact of eGDR factors on stroke occurrence. This analysis revealed that waist circumference and hypertension had a substantially more significant role in predicting incident stroke.

Our findings are consistent with previous studies on eGDR and stroke incidence. A longitudinal study of 1476 participants showed a linear relationship between eGDR and stroke incidence in people with type 2 diabetes, with reduced mortality and stroke risk associated with elevated eGDR.^9^ One of the most significant consequences of IR, which serves as the primary mechanism linking all the elements of metabolic syndrome, is patients' chronic hyperglycemia that adversely impacts brain function via a number of different pathways.^20^ IR elevates levels of extremely LDLs, which subsequently undergo conversion into residual lipoproteins that contribute to the progression of atherosclerosis. IR plays a significant role in the development of atherosclerosis by promoting a procoagulant and proinflammatory state.^6^, ^21^ Assessment of IR has emerged as a significant and extra risk factor for CVD in the assessment of mortality and chronic vascular consequences in patients with particular conditions as well as the general population.^15^, ^16^, ^17^ Considering the aggressiveness and cost of traditional methods, eGDR was developed and demonstrated to have high IR accuracy.^22^, ^23^, ^24^ Existing large‐scale nationwide studies have shown a significant correlation between IR assessed by eGDR and stroke function outcomes in patients with a first acute ischemic stroke.^25^ A higher eGDR is a good predictor of outcome, independent of traditional vascular risk factors, including diabetes, hyperlipidemia, and atrial fibrillation. Subsequently, Ren et al.'s research also showed that eGDR was positively correlated with the development of heart, stroke, and CVD.^9^ The results are consistent with the theory that eGDR may be an important predictor of CVD and a viable goal for preventing CVD in the population as a whole.

To our knowledge, only one study conducted by Ren et al. has shown that a higher estimated glomerular filtration rate (eGDR) is linked to a reduced risk of cardiovascular disease, stroke, and cardiac events in the general population. Previous studies in the general population or individuals with hypertension have only looked at one measure of eGDR and have not considered the long‐term impact of eGDR on stroke. The research was carried out in populations with cardiovascular events, and only a limited number of studies investigated the association between eGDR and the occurrence of stroke in healthy individuals. This study aimed to assess the long‐term effects of eGDR administration on stroke risk in adults with hypertension. To achieve this, we classified the level of administration into five distinct categories. In addition, our study confirmed prior research findings by demonstrating that the group with the poorest eGDR control exhibited a higher prevalence of dyslipidemia compared with the other groups. This could be the case due to the fact that IR can also change how lipids are metabolized throughout the body, which can result in dyslipidemia and the lipid triad.^6^, ^26^ This association remained constant when potential confounders were taken into account.^27^ Regular monitoring of eGDR dynamics can help detect strokes early in healthy individuals and provide new insights into clinical stroke prevention. This relationship remained significant in Model 3 of the multivariate regression analysis even after accounting for potential factors. Subgroup analysis did not find any statistically significant correlation between any category and the occurrence of stroke in patients with dyslipidemia and diabetes. Possible causes of obstructed measurement of eGDR include hypoglycemia and lipid‐lowering drugs. In subgroup analysis, a positive correlation was seen among those who did not have diabetes and dyslipidemia. Regular monitoring of eGDR dynamics can help diagnose stroke early in healthy individuals and provide new insights into clinical stroke prevention.

Finally, we use WQS regression methods to enhance the interpretability of eGDR, where we observe that waist circumference and hypertension are the main contributors to the observed defects. This is consistent with previous Xie et al.'s studies, showing that when systolic blood pressure is reduced,^7^, ^28^ the risk of stroke is significantly reduced in diabetic individuals. This is also demonstrated in the recently published ONTARGET study, where individuals with diabetes and cardiovascular risk factors experience elevated systolic blood pressure.^15^ These studies simply illustrate the significance of managing hypertension and preventing stroke. Owing to the fact that waist circumference has an effect on central obesity and is linked to IR and cardiovascular disease independently,^29^ we utilized this variable in the primary eGDR analysis. Our findings demonstrate that the risk of stroke in healthy individuals gradually rises as waist circumference increases. The question of whether obesity is a confirmed risk factor for stroke is still a subject of debate. Research has demonstrated that individuals who are overweight or obese and have type 2 diabetes experience notable and separate increases in the risk of stroke and overall death.^30^ An increase in the waist‐to‐hip ratio is also a powerful risk factor, as shown in mid‐stroke case–control studies.^13^ For obese people, this means that early lifestyle modifications and medication are required.

This work is the first known instance of utilizing cluster analysis to classify eGDR changes. Participants who exhibited superior control had the least amount of risk, while those with inferior control had the highest amount of risk. Each category represents a distinct demographic. The predictive accuracy of utilizing a single eGDR number to forecast the likelihood of a stroke has exhibited inconsistency across different cases in the past. This study establishes that individuals with a dynamic change in eGDR can be identified as being at high risk for cardiovascular disease. Furthermore, it demonstrates that such individuals can serve as therapeutically effective markers for the condition. eGDR is determined based on waist circumference, hypertension, and HbA1c levels. It is feasible to assess these biochemical indicators at the same time using a single sample, which can serve as a substitute for the hyperinsulinaemic‐hypoglycaemic method. The presence and consistent provision of the waist circumference, hypertension, and HbA1c measurements in primary‐care settings further improve the versatility and effectiveness of eGDR in clinical and epidemiological research.^31^ Second, eGDR is intended to give a more thorough assessment of IR, especially in patients with high blood pressure and a large waist circumference.

Our study had several limitations. First, while eGDR has been shown to be a surrogate marker of IR, establishing a direct link between IR and stroke requires comparison with gold‐standard diagnostic methods. This study did not make such a comparison, limiting its ability to directly explain the IR–stroke link. Second, the study included only two blood tests without comprehensive evaluation and reconstruction of eGDR. Third, the study lacks information on pharmacological treatments for secondary prevention. Some antidiabetic drugs have been shown to be beneficial to cardiovascular survival, and due to the lack of follow‐up, we cannot rule out residual confounders, which may lead to underestimation of the association of eGDR with stroke.^32^ Finally, all baseline data collected through interview questionnaires are susceptible to recall bias, which can lead to inaccurate estimates of event rates.

## 5 CONCLUSIONS

Our investigation found a correlation between inadequately regulated eGDR levels and a heightened susceptibility to stroke among middle‐aged and elderly individuals. Greater emphasis should be placed on monitoring eGDR control levels to avoid strokes, particularly in persons who do not have diabetes, dyslipidemia, and heart disease. Continuous monitoring of long‐term variations in eGDR can assist in the early identification of patients with a high risk of stroke.

## AUTHOR CONTRIBUTIONS

Hao Zhou and Jianghua Zhou conceived the study; Jiaxin Shao and Qianrong Zheng were involved in the acquisition of data; Lingzhi Ruan and Yiling Liang analyzed the data; Jiangnan Yao and Feng Zhou drafted and revised the manuscript; Fuman Cai are the guarantors of this work. The final version of the manuscript has been read and approved by all authors.

## FUNDING INFORMATION

This work was supported by the Zhejiang Provincial Health Commission (grant numbers 2024KY1272), and the funders of this study had no role in study design, data collection, data analysis, data interpretation, or report writing. The corresponding authors have full access to all data in the study and are ultimately responsible for the decision to submit for publication.

## CONFLICT OF INTEREST STATEMENT

The authors declare no conflicts of interest.

## PATIENT CONSENT STATEMENT

The patient/participant provided written informed consent to participate in this study (IRB000010052‐11,015).

## Supporting information


**Table S1.** Supporting Information.

## Data Availability

Online repositories contain the datasets used in this investigation. The names of the repositories and accession numbers can be found at http://charls.pku.edu.cn/en.
