# Supplementary material for: Association between estimated glucose disposal rate control level and stroke incidence in middle‐aged and elderly adults
Source: J Diabetes. 2024 Aug 13;16(8):e13595. doi: 10.1111/1753-0407.13595 (PMC11320750; doi:10.1111/1753-0407.13595)
Supplement: Supplementary file 1 — Table S1. Supporting Information. [file JDB-16-e13595-s001.docx]

**Table S1 Association between the cumulative average eGDR and stroke incidence**

| **Cumulative Average eGDR** | **Quintiles** | | | | | **P for trend** |
| --- | --- | --- | --- | --- | --- | --- |
|  | **Quintile 1** | **Quintile 2** | **Quintile 3** | **Quintile 4** | **Quintile 5** |  |
| **Median** | 18.42 | 23.8 | 28.7 | 31.8 | 34.5 | **-** |
| **Cases, n(%)** | 110(11.5%) | 72(7.5%) | 62(6.5%) | 33(3.4%) | 27(2.8%) | - |
| Crude, OR (95% CI) | Reference | 0.63 (0.46, 0.86) 0.003 | 0.53 (0.39, 0.74) <0.001 | 0.28 (0.18, 0.41) <0.001 | 0.22 (0.15, 0.34) <0.001 | <0.001 |
| Model 1, OR (95% CI) | Reference | 0.63 (0.46, 0.86) 0.004 | 0.56 (0.40, 0.78) <0.001 | 0.29 (0.20, 0.44) <0.001 | 0.23 (0.15, 0.36) <0.001 | <0.001 |
| Model 2, OR (95% CI) | Reference | 0.64 (0.46, 0.89) 0.007 | 0.61 (0.43, 0.89) 0.009 | 0.33 (0.21, 0.52) <0.001 | 0.27 (0.16, 0.43) <0.001 | <0.001 |
| Model 3, OR (95% CI) | Reference | 0.78 (0.56, 1.08) 0.134 | 0.78 (0.53, 1.14) 0.200 | 0.43 (0.27, 0.70) <0.001 | 0.37 (0.22, 0.62) <0.001 | <0.001 |

|  |
| --- |

eGDR**：**estimated glucose disposal rate
